# Genetic Variation in the HN and SH Genes of Mumps Viruses: A Comparison of Strains from Mumps Cases with and without Neurological Symptoms

**DOI:** 10.1371/journal.pone.0061791

**Published:** 2013-04-24

**Authors:** Aili Cui, David W. G. Brown, Wenbo Xu, Li Jin

**Affiliations:** 1 National Institute of Viral Disease Control and Prevention, Chinese Centre for Disease Control and Prevention, Beijing, People’s Republic of China; 2 Virus Reference Department, Centre for Infections, Health Protection Agency, London, United Kingdom; German Primate Center, Germany

## Abstract

**Background:**

It is known that mumps virus (MuV) strains may vary in their neurovirulent capacity, and certain MuV strains may be highly neurotropic. In animal models and epidemiological studies, mutations at specific amino acids (aa) have been proposed to be associated with neurovirulence. To assess whether these genetic variations can be observed in clinical samples from patients and if they correlate with neurovirulence as determined by clinical symptoms, 39 mumps patients with or without neurological symptoms were investigated.

**Principal Findings:**

Respiratory samples, oral fluids, throat swabs, and neurological and cerebrospinal fluid samples were tested by RT-PCR and products sequenced. Sequences of the entire small hydrophobic (SH) gene and the partial hemagglutinin-neuraminidase (HN) gene were compared.

**Conclusions:**

The results showed there was no significant difference between the samples of the two groups of patients at the aa sites in either the HN protein or the SH protein, which have previously been hypothesized to be associated with neurovirulence or antigenicity. The occurrence of neurological symptoms of mumps does not appear to be due to a single point mutation in either the HN or SH gene.

## Introduction

Mumps is caused by mumps virus (MuV) and is an acute, communicable disease transmitted via the respiratory route. The most characteristic feature of mumps is salivary gland swelling, specifically self-limiting unilateral or bilateral parotitis. MuV infection is not usually fatal, and subclinical infections often occur. However, complications of mumps include meningitis with associated deafness, pancreatitis, and orchitis, and the neurological complications can be severe. The most common neurological manifestation is aseptic meningitis, which may occur in about 10–15% of the mumps cases [1], [2].

MuV belongs to the family *Paramyxoviridae*, genus *Rubulavirus*. The single-stranded MuV genomic RNA contains seven genes: the nucleocapsid (N), phospho (P), membrane (M), fusion (F), small hydrophobic (SH), hemagglutinin-neuraminidase (HN), and large (L) protein genes. So far, 12 genotypes (A∼N, excluding E and M) have been proposed based on the diversity in the SH gene [2], [3], [4]. Some MuV strains show high neurovirulence [5], [6], [7], [8]. For example, Urabe (genotype B) and Odate-1 (genotype I) strains were both associated with a high incidence of aseptic meningitis in Japan [5]. In the 1990s, the use of the Urabe Am9 vaccine was stopped in the United Kingdom, Canada, and Japan due to its association with post-vaccination meningitis [6], [9], [10], [11]. Although most of the MuV strains of genotype A are not neurovirulent, the Kilham strain, which is a rodent brain adapted virus, is an exception and shows high neurovirulence [12]. In addition, some MuV strains within genotype C, which have two different patterns of genetic mutations, show less neurovirulence than others [8].

Previous studies have suggested that there were specific molecular markers in the MuV genome, which might be related to neurovirulence. Variations in amino acid (aa) at locations of the HN protein (aa335, 354, 356, 360, 464, and 466) and SH protein, (aa29 and aa48) were proved to be associated with MuV neurovirulence [13], [14], [15], [16], [17], [18], [19], [20], [21], and the related information is summarized in Table 1. The HN protein is also the major immunogenic protein [22], and the N-linked glycosylation sites (N-X-T or N-X-S) play important roles in maintaining the structure and antigenic properties of the extracellular domain [23]. Three epitopes (aa265∼288, aa329∼340, and aa352∼360) in the HN protein are also known to be antigenic [16], [17], [24], [25]. The mutations at several aa positions, such as aa329–340, aa354, and aa356 of the HN protein may reduce cross-neutralization capacity [17], [25], [26], [27].

**Table 1:** Amino acid changes associated with neurovirulence in the HN or SH gene.

Gene/Genome	Position ofamino acid	The changes in amino acid	Strain	Genotype orSubgenotype	Effect onneurovirulence	GenBank No.(Reference No.)	Methods
HN	335	lysine→glutamic acid (K→E)	Urabe vaccine	B	Decreased	X93181 [34]FJ375177 [39]FJ375178 [39]	Based on the epidemiological data Based on the rat model
		lysine→glutamic acid (K→E)	Urabe AM9	B	Similarneurotoxicity	[33]	Based on the rat model
		lysine→arginine (K→R)	Lit-976	C2	Decreased	AY502059 [21]	Based on the epidemiological data
		lysine→arginine (K→R)	Odate 1	I	Increased	D86170 [6]	Based on the epidemiological data
	354	proline/glutamine→histidine(P or Q→H)	Kilham	A	Increased	AY502062 [21]	Based on the epidemiological data
	356	Glutamic acid/aspartic acid→serine(E or D→S)	Kilham	A	Increased	AY502062 [21]	Based on the epidemiological data
	360	arginine→cystine (R→C)	The mutant ofKilham (M13)	A	Increased	[18]	Based on the animal model
	464	asparagine→lysine (N→K)	SKB (Urabe AM9)	B	Increased	AF314559 [12]FJ375177 [39]FJ375178 [39]	Based on the epidemiological data Based on the rat model
	466	serine→asparagine (S→N)	88-1961	H	Decreased	[22]	Based on the rat model
SH	29	alanine (A)	Lit1023	C2	Decreased	AY039721 [23]	Based on the epidemiological data
	48	serine (S)	Lit1023	C2	Decreased	AY039721 [23]	Based on the epidemiological data
Genome	at specificgenome sites	Genetic heterogeneity	Urabe AM9	B	Affect forneurovirulence.	[44]	Based on the cell line and the rat model

The reported findings were mainly observed in animal models or in vitro neural cell assays with laboratory-modified mutants, and, the importance of such aa substitutions in clinical features of MuV infection is unknown. The aim of this study was to assess whether these genetic variations could be observed in clinical samples from patients and whether they correlated with neurovirulence as determined by clinical symptoms. A total of 39 mumps patients were investigated in the study. The patients were divided into two groups: those who were reported to have neurological complications from whom a cerebrospinal fluid (CSF) sample was collected, and those who had parotitis only. MuV RNA extracts were obtained directly from clinical samples on most (31/39) of the clinical specimens and from eight MuV tissue culture fluids (TCFs) and PCR amplified. The clinical specimens included oral fluids (OF), throat swabs (TS), and CSF. The sequences of the completed SH gene,and the 459 nucleotides (nt) segment of HN gene which contained four of the nine potential N-linked glycosylation sites and most of the predicted markers of neurovirulence and antigenicity, were generated from the PCR products and analyzed by comparing the sequences between the P and N groups. In addition, the complete HN gene was also sequenced for all eight of the MuV TCFs.

## Results and Discussion

### Clinical Features of Study Subjects

The samples were divided into two groups based on the patient’s clinical symptoms (Table 2): the P group (cases 1∼22) with mumps parotitis (samples collected between 1998 and 2005), and the N group (cases 23∼39) with neurological complications and samples (collected between 2002 and 2008).

**Table 2:** Patients and specimens in the study.

Case No.	Sample type	Country	Year	Clinical group	Days after onset	Genotype
1	OF	UK	2003	P	9	H
2	OF	UK	2003	P	10	H
3	OF	UK	2004	P	6	G
4	OF	UK	2004	P	6	G
5	OF	UK	2004	P	7	G
6	OF	UK	2004	P	6	G
7	OF	UK	2004	P	8	H
8	OF	UK	2004	P	7	J
9	OF	UK	2005	P	6	G
10	OF	UK	2005	P	6	J
11	OF	UK	2005	P	6	G
12	OF	UK	2005	P	7	G
13	OF	DOM	2005	P	NK	H*
14	OF	UK	2005	P	7	J
15	OF	UK	2005	P	5	G
16	OF	UK	2005	P	5	J
17	TS	UK	2004	P	NK	C
18	TCF-TS	UK	1998	P	NK	C
19	TCF-TS	UK	2000	P	NK	C
20	TCF-NK	UK	2004	P	NK	D
21	TCF-TS	China	2005	P	NK	F
22	TCF-TS	China	2005	P	NK	F
23	CSF	UK	2004	N	8	D
24	CSF	UK	2004	N	NK	G
25	CSF	UK	2004	N	1	G
26	CSF	UK	2004	N	NK	G
27	CSF	UK	2005	N	NK	G
28	CSF	UK	2005	N	NK	G
29	CSF	UK	2005	N	6	G
30	CSF	UK	2005	N	NK	G
31	CSF	UK	2005	N	NK	G
32	CSF	UK	2005	N	NK	G
33	CSF	UK	2005	N	NK	G
34	CSF	UK	2005	N	NK	G
35	CSF	China	2004	N	NK	F
36	CSF	China	2004	N	NK	F
37	TCF-TS	China	2008	N	NK	F
38	TCF-TS	China	2008	N	NK	F
39	TCF	China	2002	N	NK	F

Note: OF, Oral fluid; CSF, cerebrospinal fluid; TS, throat swab; TCF, tissue culture fluid; P, parotitis; N, neurological complications; NK: not known; MMR, measles-mumps-rubella vaccine; UK, United Kingdom.

* Dominican Republic linked re-infection case in Sweden, childhood infected (Dr. Kari Johansen, Swedish Institute for Infectious Disease Control).

### Genetic Correlation of the MuV Strains

The 39 specimens investigated were all found to be positive by RT-PCR based on the previously published assay for the entire SH gene [28] and the newly developed assay for the partial HN gene. The nucleotide sequences of the SH gene (JQ034428∼ JQ034456), the partial HN gene (JQ034467∼JQ034493, JQ034499∼JQ034501, and JQ034503), and the entire HN gene (JQ034459∼JQ034466) were submitted to GenBank.

The 39 MuV strains belonged to seven different genotypes, namely, H, G, J, C, D, and F in group P, and D, G, and F in group N [3]. The results showed that genotype G and F strains generated from case 24–39 (Table 2) were also associated with neurovirulence in addition to genotypes C, D, H, J, and I, as reported previously [7], [8].

The phylogenetic tree was constructed based on the 459 nt of the HN gene, which contained most of the neurovirulence-related sites. The phylogenetic analysis also enabled the assignment of the study sequences to seven genotypes, which was similar to the results based on the SH gene (Table 2).

To find the mutations associated with neurovirulence at the specific aa, the sequences of the SH and HN genes from the same genotype were compared between group P and N. Identical sequences were found in both the SH and HN genes obtained from the two specimen types, OF from the group P patients and CSF from the group N patients. Within genotype G, cases 9 and 15 from the OF samples of group P were 100% identical to cases 26, 28, and 33 from the CSF samples of group N, based on the SH sequences. Similarly, the sequences of cases 9, 11, 12, and 15 obtained from the OF samples of group P were identical to cases 25, 26, 28, 29, 30, and 33 obtained from the CSF samples of group N, based on the partial HN gene. All these samples were collected sporadically in the UK during the mumps endemic period between 2004 and 2005, and belonged to genotype G without any epidemiological links between the cases. Within genotype D, both the SH and HN sequences from the CSF sample case 23 collected from a patient of group N in Cardiff were identical to those from a cell culture sample, case 20/group P in Nottingham 2 months later, suggesting that identical sequences could be found from patients with or without neurological complications. Thus, there was no evidence to support the hypothesis that genomic variations in these SH and HN regions that contain the proposed functional mutations directly affect viral neuropathogenicity in patients.

For a more comprehensive analysis of neurovirulence sites in the HN protein, the eight TCF isolates, five from the P group and three from the N group were sequenced for the entire HN gene (1749 nt) and analyzed with other HN sequences downloaded from GenBank. These sequences were obtained from patients with or without neurological symptoms. The five TCF isolates of the P group belonged to three genotypes, including two of genotype C, one of genotype D, and two of genotype F. The three TCF isolates of the N group were all genotype F. For the eight TCF isolates, there were 149 nt/33 aa mutations in the entire HN protein (1749 nt/583 aa), and most of them were associated with genotypes or specificity of the strains. By comparing the sequences of the entire HN gene, no significant difference was found, which may be associated with MuV neurovirulence in the P and N groups.

### Genetic Analysis of the Neuropathogenic Sites

**Figure 1:**
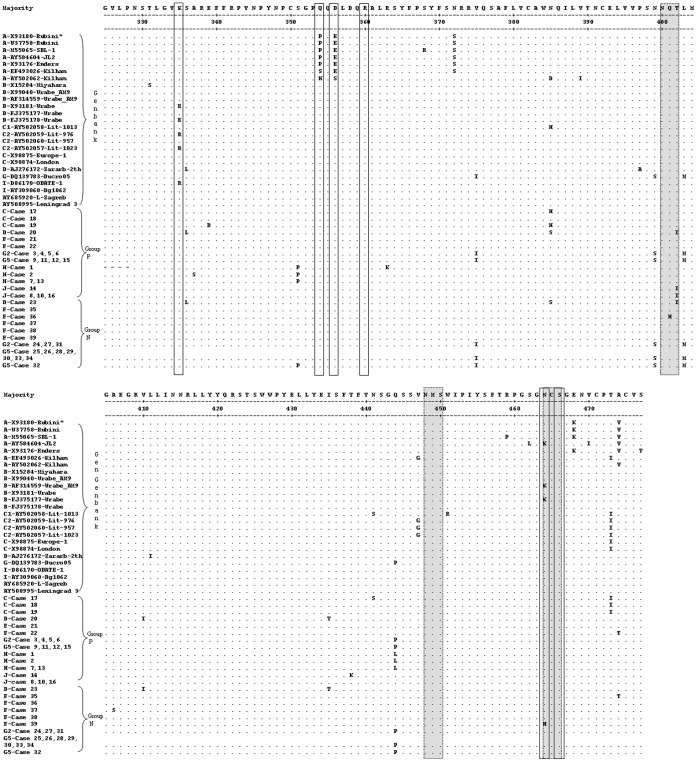
Alignment of the inferred partial aa sequences of the HN gene. The potential glycosylation sites are shown in shadow and the potential neurovirulence sites are marked in boxes. Period (.) denotes identity with the uppermost sequence; (-) denotes areas that were not determined; (*) represents Genotype-strain ID.

**Figure 2:**
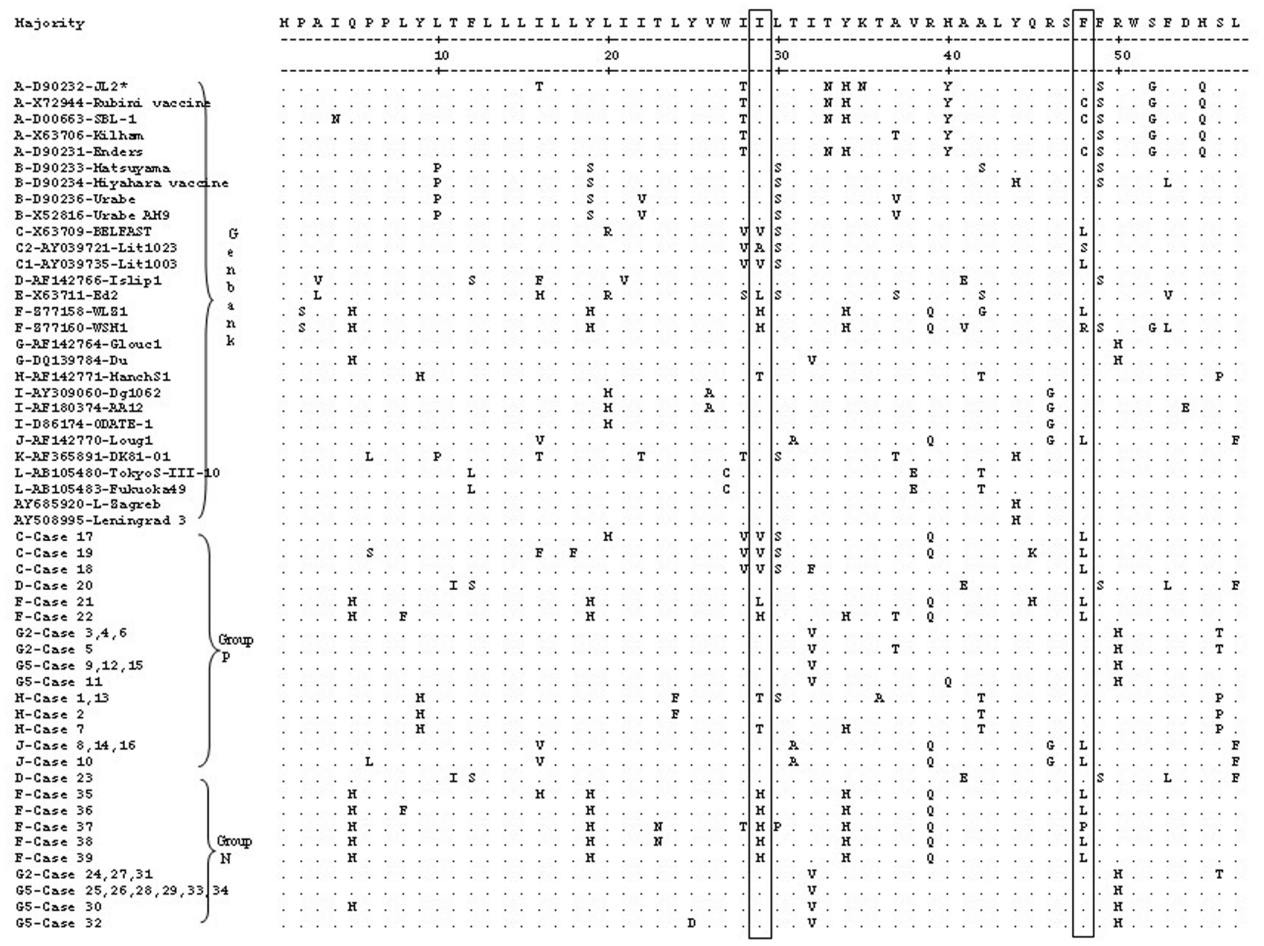
Alignment of the inferred aa sequences of the SH gene. The potential neurovirulence sites are marked in boxes. Period (.) denotes identity with the majority sequence; (-) denotes areas that were not determined; (*) represents Genotype-strain ID.

Some literatures have demonstrated that single aa mutations could have dramatic effects on virulence/attenuation. For example, in each of the three Sabin vaccine strains of poliovirus, there were only a small number of point mutations that seemed to give rise to their attenuation phenotypes [29]. It is known that MuV strains may vary in their neuropathogenic capacity and that certain virus strains may cause a relatively higher frequency of meningitis cases than the others. The aa335, 354, 356, 360, 464, and 466 in the HN protein and the aa29 and 48 in the SH protein were hypothesized to be associated with neurovirulence. However, such findings associated with MuV neuropathogenicity were not clinically investigated in mumps patients. In this study, the two groups of MuV patients, with (group N) or without (group P) neurological symptoms were examined, and the sequences of the partial HN and SH genes were compared between the P and N groups. Figure 1 shows the alignment of the 39 sequences obtained from the study samples and 25 sequences from GenBank based on the 153 aa of the partial HN protein. The boxed aa335, 354, 356, 360, 464, and 466 in Figure 1 are suspected to be the neurovirulent sites. To prevent the impacts from mutations caused by genotype, the sequences from the clinical specimens were compared within the same genotype. The mutations in the sequences obtained in the P and N groups showed no noticeable tendency which could be associated with neurovirulence. Furthermore, based on the aa57 of the SH protein, the alignment of the 39 sequences from the study and 28 sequences from GenBank is shown in Figure 2. Similarly, no significant difference was found in the boxed aa29 and 48, which were suggested as attenuation markers for neuropathogenic capacity [21]. Most of the mutations were associated with the assigned genotypes of the MuV strains.

Adenine/lysine at the 1081 nt (aa335) in some of the mumps vaccines and wild-type strains was found to be associated with mumps neurological complications, whereas the presence of guanine/glutamic acid at this position was associated with a neuroattenuated phenotype [13], [16], [30]. For example, the Urabe AM9 vaccine is a mixture of MuVs that differ at a single codon of the HN gene; the parental-type sequence has an A residue at 1081 nt and encodes lysine at aa335, whereas the variant sequence has a G residue at 1081 nt that encodes glutamic acid. Such Glu^335^→Lys^335^ reversion in the HN protein was detected in the vaccine strains isolated from recent vaccinated individuals with or without mumps meningitis [30]. MuV homogeneous to the variant Glu^335^ form of the HN gene may be safer than the original Urabe AM9 vaccine. On the other hand, two variants of genotype C viruses isolated from the mumps epidemic in Lithuania between 1998 and 2000 were found to cause different symptoms in patients [21]. The patients who were infected by the C1 variant suffered from meningitis, whereas the patients infected by the C2 variant suffered from parotitis only. A particularly striking difference between C1 and C2 viruses was also aa335 in the HN protein; lysine was generally found in the neurovirulent C1 strains, whereas arginine was observed in the non-neurovirulent C2 strains (Figure 1). The change from lysine to arginine in the C2 strains attenuated neuropathogenesis [19], [21].

In addition, the Odate-1 strain with documented high neurovirulence was found to have arginine at aa335, which showed that virus strains with amino acids other than lysine at this position might also be associated with high neurovirulence [5], [31].

The above-mentioned studies (Table 1) suggest that variation at the 1081 nt (aa335) of the HN protein is associated with the neurovirulent phenotype. However, it is also found that other virus strains with presumed low neurovirulence, such as strains Enders, Rubini, Jeryl Lynn, RW, and SBL-1, all have lysine at aa335 [32]. And other literatures have reported that the recombinant Urabe Am9 MuVs that were rescued only differed at aa335 in the HN protein. Regardless of the presence of lysine or glutamic acid at aa335, the rescued viruses exhibited strong neurovirulence in the rat model [33]. Presence of lysine at aa 335 of the hemagglutinin-neuraminidase protein of MuV vaccine strain Urabe AM9 is not a requirement for neurovirulence, suggesting that this mutation alone is not critical for MuV neurovirulence. Similarly, in this study, all the 39 sequences from MuV patients belonging either to group P or N were found to have lysine at aa335 of the HN gene (Figure 1). So it is not entirely certain that this mutation alone is responsible for a change in neurovirulence. Nevertheless, the importance of this mutation in the virus virulence cannot be ignored because most of the MuV isolates possess lysine at aa335. Some works have shown that lysine at aa335 increase the virus affinity for cell receptors and enzymatic activity [34], [35].

Most of the MuV strains of genotype A are not neuropathogenic, but the Kilham strain, which was isolated from human breast milk and serially passaged in suckling hamster brain, formed an exception with its high neuropathogenic capacity [12]. In the HN protein of the Kilham strain (GB. AY502062), histidine and serine were found at aa354 and 356, which might be associated with mumps neurovirulence [20]. Such changes, however, were not found in any strain investigated in this study (Figure 1), except for proline and glutamic acid, which were at aa354 and 356, respectively, of some historic strains, which belonged to genotype A (Figure 1).

A change (arginine→cysteine) at aa360 of the highly neurovirulent hamster brain-adapted Kilham strain was associated with a decreased ability to infect neurons in hamsters [17]; a replacement at aa464 (asparagine→lysine) of the Urabe AM9 (AF314559) vaccine strain was also associated with causing meningitis in vaccine recipients [13], [14], [30]. Strain 88-1961, a wild-type strain isolated from a patient with neurological symptoms, caused severe, devastating neurological damage (e.g., hydrocephalus) in the rat neurovirulence assay [12]. The serine→asparagine substitution at aa466 in the HN of strain 88-1961 (Table 1), which is predicted to result in a loss of N glycosylation site (aa464 to aa466) had the potential to affect virus tropism and virulence, and was associated with an aa change in the L protein with MuV neuroattenuation [20], [36], [37]. However, in the present study, the above-mentioned substitutions in the HN gene were not found in the 39 sequences generated in the P and N groups. JL2 vaccine strain (AY584604) was found to be associated with low neurovirulence. Leningrad-3 vaccine and L-Zagreb vaccine are known to cause adverse events of neurovirulence. The HN sequences of these vaccine strains were also included for analysis in the present study. Similarly, no mutation that may be associated with neurovirulence was found in the sequences [38], [39].

With regard to the SH protein, the combination of alanine at aa29 with serine at aa48 was also suspected to be the molecular marker of attenuation of the neuropathogenic capacity [21]. The genotype C2 virus (Lit1023, AY039721) appeared to be less neuropathogenic than the genotype C1. The C1 virus strains, e.g. Lit1003 (AY039735), contained valine and leucine at aa29 and aa48, respectively (Figure 2). This combination of mutations was detected in some strains investigated in this study, such as cases 17, 18, and 19, which all belonged to the P group and were all assigned to genotype C. The results again showed that mutations in the SH gene were associated with the assigned genotypes of MuV, rather than the clinical symptoms (Figure 2).

The SH gene is the gene that displays the highest degree of heterogeneity among different isolates and genotypes, which is the reason for the genotyping to be mainly based on the differences in the SH gene. Importantly, however, it has been known for a long time that the SH gene is dispensable for efficient virus growth in vitro, and recent evidence from a rat model for MuV neurovirulence strongly suggests that the SH gene does not play a critical role in neurovirulence in vivo [40]. Thus, the mutations found in the SH are unlikely to be associated with neurovirulence and are merely a reflection of the high mutation rate seen in this gene. The fact that the virus tolerates such a high mutation rate is also an indication that the SH gene is a luxury gene that does not play a pivotal role in the virus’ lifecycle.

In this study, only the SH gene and the partial HN gene were analyzed for the neurovirulence sites due to the limitation of the clinical specimens. However, there is a possibility that genes other than the HN and SH genes might harbor important mutations associated with MuV neurovirulence. The recent progress in the field suggests that mutations in other genes such as the V gene [41] or the L gene [37] might be critical for virus attenuation. Moreover, recent studies using genetically modified MuVs and suitable animal models suggest that genetic determinants of MuV neurovirulence are strain-specific. The major genetic determinant of neurovirulence of the rodent-adapted Kilham strain appears to be the F gene [42]. While the neurovirulence of the wild-type MuV isolate 88-1961 seems to be a multigenic trait [43]. Such a scenario was described with the 88-1961 strain, where an additive effect on neuroattenuation of the two mutations – one in the HN protein and one in the L polymerase protein – was demonstrated [37]. Thus, although a common mutation in the 39 samples, which might be associated with neurovirulence, was not observed, the possibility that any of the mutations found may be associated with unidentified mutations located on other genes, which indeed play a role in neurovirulence, cannot be ruled out. In addition, Sauder et al previously demonstrated that a neuroattenuated strain of Urabe Am9 displayed lysine at aa335 in the HN protein [44]. Despite a dramatic decrease in virus virulence (as assayed in rats), the only genomic changes were in the form of changes in the level of genetic heterogeneity at specific genome sites, i.e., either selection of one nucleotide variant at positions where the starting material exhibited nucleotide heterogeneity or the evolution of an additional nucleotide to create a heterogenic site. This finding suggests that changes in the level of genetic heterogeneity at specific genome sites can have profound neurovirulence phenotypic consequences.

### Genetic Analysis of the Antigenic Domains within the Partial HN Gene

All predictions for MuV HN are inferred from the model of NDV-HN, and the potential glycosylation sites in the HN protein play roles in maintaining the structural and antigenic properties of the extracellular domain [23]. In this study, four of the nine potential N-linked glycosylation sites (N-X-T or N-X-S) were found in this 153 aa of the HN fragment amplified by the newly developed PCR assay, which were aa329∼331, 400∼402, 448∼450, and 464∼466 (Figure 1) The sequence analyses showed that the N-linked glycosylation site at aa400∼402 disappeared due to a change from tyrosine to isoleucine at aa402 in four OF samples (cases 8, 10, 14, and 16 belonging to group P), one CSF sample (group N, case 23), and one cell culture sample (group P, case 20). Among these six samples, the four OF samples belonged to genotype J and the others belonged to genotype D. The N-glycosylation site at aa464∼466 was destroyed in vaccine strains JL2 (AY584604) and Urabe AM9 (AF314559) belonging to genotype A and B, respectively, because of the asparagine to lysine change at aa464, as well as in the HN sequence of case 39 belonging to genotype F (group N), because of the asparagine to histidine change at aa464. These results (Figure 1) suggest that mutations occurring at the N-glycosylation sites could be random and not clearly associated with either genotype classification or virus neurovirulence.

According to the previous reports, mutations at several aa positions, such as aa329∼340, 352, 354∼356, and 360 of the HN gene may reduce cross-neutralization capacity [17], [25], [26], [27]. Two of the three epitopes (aa265∼288, 329∼340, and 352∼360) known to be antigenic [17], [24], [25], [26] were located within the partial sequences of the HN protein. Based on the inferred aa sequences, there was no variation found at these previously proposed antigenic domains in the sequences, which may be significantly associated with neurovirulence or antigenicity of these MuVs. The mutations occurring at the N-glycosylation sites and those particular aa were mainly associated with genotype classification between P and N groups.

### Conclusion

In this study, the 39 sequences were directly obtained from clinical specimens or less than three passages of the isolates, avoiding the potential impact on genetic variation during viral propagation in cell cultures. Based on the analysis of the 39 sequences in this study, it was found that there was no significant difference in the predicted neurovirulence sites among the sequences in the P and N groups. Therefore, the contribution of those random changes detected in the specimens to neurovirulence has been not confirmed by this study. This is not surprising because it is unlikely that any single nucleotide or aa could be completely responsible for the overall virulence or attenuation of a specific virus strain, particularly for RNA viruses [45]. Moreover, no significant mutation was found at the previously proposed antigenic domains in all the sequences generated in the P and N groups in the present study. The results showed limited evidence that such mutations contribute to MuV neurovirulence. However, the occurrence of mumps neurological symptoms could not be simply due to the mutation in any single nucleotide or aa position in the sequence of the HN or SH protein of MuV, which maybe in agreement with the observation of unidentified mutations located on other genes, that, indeed, play a role in neurovirulence. Thus, whilst it is fair to conclude that no single mutation found in the sequenced HN protein segment is sufficient to induce neurovirulence, additive and/or synergistic effects of other mutations,- either on the same protein or on others, cannot be dismissed.

## Materials and Methods

### Patients and Specimens

A total of 31 clinical specimens and eight TCFs were obtained from 39 mumps patients during the period of 1998–2008 in three countries, seven from China, one from Sweden (link to Dominican Republic), and 31 from the UK. The 39 patients were clinically diagnosed with mumps infection and some of these patients were also laboratory-confirmed by their serological status. Mumps IgM/IgG capture EIA kits (Microimmune, UK) were used at HPA, UK, and the Serion ELISA classic Mumps Virus IgM kit (Institute Virion\Serion GmbH, Germany) was used at China CDC.

Based on the clinical symptoms, these patients were divided into two groups. The 17 patients with neurological symptoms, such as headache, neck stiffness, and photophobia, were classified as the N group, and the 22 patients who showed either uni- or bilateral parotitis without neurological symptoms were defined as the P group (Table 2).

The 39 specimens included 16 OF, one TS, and five TCFs from the 22 patients in group P; and 14 CSF and three TCFs from the 17 patients in group N. All eight TCFs containing suspected primary mumps isolates were originally from patients’ TS specimens, except for one (case 20), for which the specimen type was not known. These primary mumps isolates were harvested after two or three passages in either Vero or Vero-SLAM cells according to the standard operating procedure (HPA, UK).

### RNA Extraction and RT-PCR

Total nucleic acids were extracted using either the silica-guanidinium thiocyanate method for specimens collected before 2003 or MagNAPure total nucleic acid extractor (Roche, UK). The RNA was reverse-transcribed using Moloney murine leukemia virus reverse transcriptase and random hexamers, as described previously [46], [47]. A total of 20 µl of cDNA were added to the first-round PCR, and 10 µl of the first-round PCR amplicon were then used as template for nested PCR. The nested PCR for the HN gene was carried out with two sets of primers, primers MuV-HN1 (5′-GCTACTTTGGTGCCAGGAG-3′, nt: 982–1000) and MuV-HN2r (5′-GGAGTTAATGGCCAGGGAT-3′, nt: 1523–1541), each at a concentration of 10 pmol, for the first-round PCR, and MuV-HN3 (5′-TGATCTTTCCTGCATATGG-3′, nt: 1031–1049) and MuV-HN4r (5′-GCCAGGGATCAAGATAAAC-3′, nt: 1513–1531), at a concentration of 25 pmol, for the nested PCR. The nucleotide positions were according to the sequence of the HN gene of MuV isolate 4829 [GenBank accession number AF448527]. PCR amplification for the HN gene was carried out as follows: an initial incubation for 2 min at 95°C; 25 cycles of 1 min at 95°C, 1.5 min at either 50°C (for the first round) or 55°C (for the second round), and 2 min at 72°C; and a final extension step for 5 min at 72°C; similar steps were employed for the PCR amplification of the SH gene. The PCR amplification for the TCF isolates was carried out by employing the first-round only. A similar process was performed for the SH gene, with the 598-nt region including the entire SH gene, as described in the previous reports [28]. The complete coding region of the HN gene was amplified as described previously [48].

The positive and negative controls and the strict partitions of laboratory were applied thoroughly in the study according to the HPA SOPs.

### Nucleotide Sequencing and Analysis

The PCR products of the partial HN gene, entire HN gene, and SH gene were directly purified with the QIAquick PCR purification kit (Qiagen, UK). The purified fragments were bi-directionally sequenced with primers for the partial HN gene, entire HN gene, and SH gene. The sequences were commercially generated from the PCR products with the DyeDeoxy terminator sequencing kit (Applied Biosystems, UK), as described previously [28], [48]. The sequence data were stored as standard chromatogram format (.ab1) files and were analyzed with Sequencher software (version 4.0.5; GeneCodes, Ann Arbor, MI).The nucleotide sequences and the inferred aa sequences were analyzed using the Clustal program within the Megalign suite of DNASTAR software (Lasergene). To identify the genotypes, the divergences between the MuV sequences were calculated based on both the 459 nt HN gene and the SH gene, and the phylogenetic trees were drawn by bootstrap analysis using the Neighbor-Joining algorithm implemented within the MEGA software package (version 4.0).

### Ethics Statement

This study did not directly involve human participants or human experimentation. The human samples used in this study were collected by the hospitals or clinicians for the laboratory diagnosis of mumps. Samples were then stored in UK HPA and China CDC after detection respectively. All the specimens were unlinked from the patient identifiers before analysis. This study of mumps was approved by the Ethic Review Committee of the Chinese CDC. Ethical approval was not required in the UK, and samples were handled following the UK guidance on the use of clinical samples.

## References

[1] Hviid A, Rubin S, Muhlemann K (2008) Mumps. Lancet 371: 932–944. 18342688 10.1016/S0140-6736(08)60419-5

[2] World Health Organization (2012) Mumps virus nomenclature update: 2012. Weekly Epidemiological Record (WER) 87: 217–224.24340404

[3] Jin L, Rima B, Brown D, Örvell C, Tecle T, et al (2005) Proposal for genetic characterisation of wild-type mumps strains: preliminary standardisation of the nomenclature. Arch Virol 150: 1903–1909. 15959834 10.1007/s00705-005-0563-4

[4] Santos CL, Ishida MA, Foster PG, Sallum MA, Benega MA, et al (2008) Detection of a new mumps virus genotype during parotitis epidemic of 2006–2007 in the state of Sao Paulo, Brazil. J Med Virol 80: 323–329. 18098149 10.1002/jmv.21068

[5] Saito H, Takahashi Y, Harata S, Tanaka K, Sano T, et al (1996) Isolation and characterization of mumps virus strains in a mumps outbreak with a high incidence of aseptic meningitis. Microbiol Immunol 40: 271–275. 8709862 10.1111/j.1348-0421.1996.tb03346.x

[6] Sugiura A, Yamada A (1991) Aseptic meningitis as a complication of mumps vaccination. Pediatr Infect Dis J 10: 209–213. 2041668 10.1097/00006454-199103000-00008

[7] Tecle T, Johansson B, Jejcic A, Forsgren M, Örvell C (1998) Characterization of three co-circulating genotypes of the small hydrophobic protein gene of mumps virus. J Gen Virol 79 (Pt 12): 2929–2937. 10.1099/0022-1317-79-12-2929 9880006

[8] Tecle T, Bottiger B, Örvell C, Johansson B (2001) Characterization of two decades of temporal co-circulation of four mumps virus genotypes in Denmark: identification of a new genotype. J Gen Virol 82: 2675–2680. 11602779 10.1099/0022-1317-82-11-2675

[9] Furesz J, Contreras G (1990) Vaccine-related mumps meningitis–Canada. Can Dis Wkly Rep 16: 253–254. 2285950

[10] Miller E, Goldacre M, Pugh S, Colville A, Farrington P, et al (1993) Risk of aseptic meningitis after measles, mumps, and rubella vaccine in UK children. Lancet 341: 979–982. 8096942 10.1016/0140-6736(93)91069-x

[11] Ueda K, Miyazaki C, Hidaka Y, Okada K, Kusuhara K, et al (1995) Aseptic meningitis caused by measles-mumps-rubella vaccine in Japan. Lancet 346: 701–702. 10.1016/s0140-6736(95)92311-x 7658837

[12] Rubin SA, Pletnikov M, Taffs R, Snoy PJ, Kobasa D, et al (2000) Evaluation of a neonatal rat model for prediction of mumps virus neurovirulence in humans. J Virol 74: 5382–5384. 10799619 10.1128/jvi.74.11.5382-5384.2000 PMC110897

[13] Afzal MA, Yates PJ, Minor PD (1998) Nucleotide sequence at position 1081 of the hemagglutinin-neuraminidase gene in the mumps Urabe vaccine strain. J Infect Dis 177: 265–266. 9419205 10.1086/517353

[14] Amexis G, Fineschi N, Chumakov K (2001) Correlation of genetic variability with safety of mumps vaccine Urabe AM9 strain. Virology 287: 234–241. 11504558 10.1006/viro.2001.1009

[15] Brown EG, Wright KE (1998) Genetic studies on a mumps vaccine strain associated with meningitis. Rev Med Virol 8: 129–142. 10398501 10.1002/(sici)1099-1654(199807/09)8:3<129::aid-rmv213>3.0.co;2-z

[16] Cusi MG, Santini L, Bianchi S, Valassina M, Valensin PE (1998) Nucleotide sequence at position 1081 of the hemagglutinin-neuraminidasegene in wild-type strains of mumps virus is the most relevant marker of virulence. J Clin Microbiol 36: 3743–3744. 9867495 10.1128/jcm.36.12.3743-3744.1998 PMC105283

[17] Kovamees J, Rydbeck R, Örvell C, Norrby E (1990) Hemagglutinin-neuraminidase (HN) amino acid alterations in neutralization escape mutants of Kilham mumps virus. Virus Res 17: 119–129. 1705373 10.1016/0168-1702(90)90073-k

[18] Mori C, Tooriyama T, Imagawa T, Yamanishi K (1997) Nucleotide sequence at position 1081 of the hemagglutinin-neuraminidase gene in the mumps virus Urabe vaccine strain. J Infect Dis 175: 1548–1549. 9180205 10.1086/516499

[19] Rafiefard F, Johansson B, Tecle T, Örvell C (2005) Characterization of mumps virus strains with varying neurovirulence. Scand J Infect Dis 37: 330–337. 16051568 10.1080/00365540510031674

[20] Rubin SA, Amexis G, Pletnikov M, Li Z, Vanderzanden J, et al (2003) Changes in mumps virus gene sequence associated with variability in neurovirulent phenotype. J Virol 77: 11616–11624. 14557647 10.1128/JVI.77.21.11616-11624.2003 PMC229304

[21] Tecle T, Mickiene A, Johansson B, Lindquist L, Örvell C (2002) Molecular characterisation of two mumps virus genotypes circulating during an epidemic in Lithuania from 1998 to 2000. Arch Virol 147: 243–253. 11890522 10.1007/s705-002-8317-y

[22] Carbone KM, Rubin S (2007) Mumps Virus; Knipe D HP, Griffin D, Lamb R, Martin M. Lippincott Williams, editor.

[23] Crennell S, Takimoto T, Portner A, Taylor G (2000) Crystal structure of the multifunctional paramyxovirus hemagglutinin-neuraminidase. Nat Struct Biol 7: 1068–1074. 11062565 10.1038/81002

[24] Kulkarni-Kale U, Ojha J, Manjari GS, Deobagkar DD, Mallya AD, et al (2007) Mapping antigenic diversity and strain specificity of mumps virus: a bioinformatics approach. Virology 359: 436–446. 17081582 10.1016/j.virol.2006.09.040

[25] Örvell C, Kalantari M, Johansson B (1997) Characterization of five conserved genotypes of the mumps virus small hydrophobic (SH) protein gene. J Gen Virol 78 (Pt 1): 91–95. 10.1099/0022-1317-78-1-91 9010290

[26] Cusi MG, Fischer S, Sedlmeier R, Valassina M, Valensin PE, et al (2001) Localization of a new neutralizing epitope on the mumps virus hemagglutinin-neuraminidase protein. Virus Res 74: 133–137. 11226581 10.1016/s0168-1702(00)00254-9

[27] Örvell C, Tecle T, Johansson B (2002) Mumps virus: an underestimated viral pathogen. Current Topics in Virol 2: 100–113.

[28] Jin L, Beard S, Brown DW (1999) Genetic heterogeneity of mumps virus in the United Kingdom: identification of two new genotypes. J Infect Dis 180: 829–833. 10438373 10.1086/314957

[29] Minor PD, Macadam AJ, Stone DM, Almond JW (1993) Genetic basis of attenuation of the Sabin oral poliovirus vaccines. Biologicals 21: 357–363. 8024751 10.1006/biol.1993.1096

[30] Brown EG, Dimock K, Wright KE (1996) The Urabe AM9 mumps vaccine is a mixture of viruses differing at amino acid 335 of the hemagglutinin-neuraminidase gene with one form associated with disease. J Infect Dis 174: 619–622. 8769623 10.1093/infdis/174.3.619

[31] Saika S, Kidokoro M, Ohkawa T, Aoki A, Suzuki K (2002) Pathogenicity of mumps virus in the marmoset. J Med Virol 66: 115–122. 11748667 10.1002/jmv.2119

[32] Yates PJ, Afzal MA, Minor PD (1996) Antigenic and genetic variation of the HN protein of mumps virus strains. J Gen Virol 77 (Pt 10): 2491–2497. 10.1099/0022-1317-77-10-2491 8887482

[33] Sauder CJ, Zhang CX, Link MA, Duprex WP, Carbone KM, et al (2009) Presence of lysine at aa 335 of the hemagglutinin-neuraminidase protein of mumps virus vaccine strain Urabe AM9 is not a requirement for neurovirulence. Vaccine 27: 5822–5829. 19660591 10.1016/j.vaccine.2009.07.051

[34] Santos-Lopez G, Scior T, Borraz-Arguello Mdel T, Vallejo-Ruiz V, Herrera-Camacho I, et al (2009) Structure-function analysis of two variants of mumps virus hemagglutinin-neuraminidase protein. Braz J Infect Dis 13: 24–34. 19578626 10.1590/s1413-86702009000100007

[35] Reyes-Leyva J, Banos R, Borraz-Arguello M, Santos-Lopez G, Rosas N, et al (2007) Amino acid change 335 E to K affects the sialic-acid-binding and neuraminidase activities of Urabe AM9 mumps virus hemagglutinin-neuraminidase glycoprotein. Microbes Infect 9: 234–240. 17223599 10.1016/j.micinf.2006.11.011

[36] Malik T, Wolbert C, Mauldin J, Sauder C, Carbone KM, et al (2007) Functional consequences of attenuating mutations in the haemagglutinin neuraminidase, fusion and polymerase proteins of a wild-type mumps virus strain. J Gen Virol 88: 2533–2541. 17698664 10.1099/vir.0.82935-0

[37] Malik TH, Wolbert C, Nerret L, Sauder C, Rubin S (2009) Single amino acid changes in the mumps virus haemagglutinin-neuraminidase and polymerase proteins are associated with neuroattenuation. J Gen Virol 90: 1741–1747. 19282431 10.1099/vir.0.009449-0

[38] Arruda WO, Kondageski C (2001) Aseptic meningitis in a large MMR vaccine campaign (590,609 people) in Curitiba, Parana, Brazil, 1998. Rev Inst Med Trop Sao Paulo 43: 301–302. 11696855 10.1590/s0036-46652001000500012

[39] Cizman M, Mozetic M, Radescek-Rakar R, Pleterski-Rigler D, Susec-Michieli M (1989) Aseptic meningitis after vaccination against measles and mumps. Pediatr Infect Dis J 8: 302–308. 2726323

[40] Malik T, Shegogue CW, Werner K, Ngo L, Sauder C, et al (2011) Discrimination of mumps virus small hydrophobic gene deletion effects from gene translation effects on virus virulence. J Virol 85: 6082–6085. 21471236 10.1128/JVI.02686-10 PMC3126307

[41] Xu P, Luthra P, Li Z, Fuentes S, D’Andrea JA, et al (2012) The V protein of mumps virus plays a critical role in pathogenesis. J Virol 86: 1768–1776. 22090137 10.1128/JVI.06019-11 PMC3264346

[42] Lemon K, Rima BK, McQuaid S, Allen IV, Duprex WP (2007) The F gene of rodent brain-adapted mumps virus is a major determinant of neurovirulence. J Virol 81: 8293–8302. 17475640 10.1128/JVI.00266-07 PMC1951292

[43] Sauder CJ, Zhang CX, Ngo L, Werner K, Lemon K, et al (2011) Gene-specific contributions to mumps virus neurovirulence and neuroattenuation. J Virol 85: 7059–7069. 21543475 10.1128/JVI.00245-11 PMC3126569

[44] Sauder CJ, Vandenburgh KM, Iskow RC, Malik T, Carbone KM, et al (2006) Changes in mumps virus neurovirulence phenotype associated with quasispecies heterogeneity. Virology 350: 48–57. 16494912 10.1016/j.virol.2006.01.035

[45] Domingo E, Holland JJ (1997) RNA virus mutations and fitness for survival. Annu Rev Microbiol 51: 151–178. 9343347 10.1146/annurev.micro.51.1.151

[46] Jin L, Richards A, Brown DW (1996) Development of a dual target-PCR for detection and characterization of measles virus in clinical specimens. Mol Cell Probes 10: 191–200. 8799373 10.1006/mcpr.1996.0027

[47] Jin L, Brown DW, Litton PA, White JM (2004) Genetic diversity of mumps virus in oral fluid specimens: application to mumps epidemiological study. J Infect Dis 189: 1001–1008. 14999602 10.1086/382134

[48] Jin L, Beard S, Hale A, Knowles W, Brown DW (2000) The genomic sequence of a contemporary wild-type mumps virus strain. Virus Res 70: 75–83. 11074127 10.1016/s0168-1702(00)00211-2

